# *In vivo* imaging xenograft models for the evaluation of anti‐brain tumor efficacy of targeted drugs

**DOI:** 10.1002/cam4.1255

**Published:** 2017-11-10

**Authors:** Kenji Kita, Sachiko Arai, Akihiro Nishiyama, Hirokazu Taniguchi, Koji Fukuda, Rong Wang, Tadaaki Yamada, Shinji Takeuchi, Shoichiro Tange, Atsushi Tajima, Mitsutoshi Nakada, Kazuo Yasumoto, Yoshiharu Motoo, Takashi Murakami, Seiji Yano

**Affiliations:** ^1^ Division of Medical Oncology Kanazawa University Cancer Research Institute Kanazawa Japan; ^2^ Department of Respiratory Medicine Nagasaki University Graduate School of Biomedical Sciences Nagasaki Japan; ^3^ Department of Pulmonary Medicine Graduate School of Medical Science Kyoto Prefectural University of Medicine Kyoto Japan; ^4^ Department of Bioinformatics and Genomics Graduate School of Advanced Preventive Medical Sciences Kanazawa University Kanazawa Japan; ^5^ Department of Neurosurgery Graduate School of Medical Science Kanazawa University Kanazawa Japan; ^6^ Department of Medical Oncology Kanazawa Medical University Kahoku Japan; ^7^ Department of Microbiology Faculty of Medicine Saitama Medical University Saitama Japan

**Keywords:** Brain tumor, entrectinib, *NTRK1*, osimertinib, TRK‐A

## Abstract

Molecular‐targeted drugs are generally effective against tumors containing driver oncogenes, such as *EGFR*,*ALK*, and *NTRK1*. However, patients harboring these oncogenes frequently experience a progression of brain metastases during treatment. Here, we present an *in vivo* imaging model for brain tumors using human cancer cell lines, including the *EGFR*‐L858R/T790M‐positive H1975 lung adenocarcinoma cells, the NUGC4 hepatocyte growth factor (HGF)‐dependent gastric cancer cells, and the KM12SM colorectal cancer cells containing the *TPM3‐NTRK1* gene fusion. We investigated the efficacy of targeted drugs by comparison with their effect in extracranial models. *In vitro*, H1975 cells were sensitive to the third‐generation epidermal growth factor receptor inhibitor osimertinib. Moreover, HGF stimulated the proliferation of NUGC4 cells, that was inhibited by crizotinib, which has anti‐MET activity. KM12SM cells were sensitive to the tropomyosin‐related kinase‐A inhibitors crizotinib and entrectinib. In *in vivo* H1975 cell models, osimertinib inhibited the progression of both brain and subcutaneous tumors. Furthermore, in *in vivo* NUGC4 cell models, crizotinib remarkably delayed the progression of brain tumors, and that of peritoneal carcinomatosis. Interestingly, in *in vivo* KM12SM cell models, treatment with crizotinib delayed the progression of liver metastases, but not that of brain tumors. Conversely, treatment with entrectinib discernibly delayed the progression of both tumor types. Thus, the effect of targeted drugs against brain tumors can differ from the one reported in extracranial tumors. Moreover, the same multikinase inhibitory drug can display different efficacies in brain tumor models containing different drivers. Therefore, our *in vivo* imaging model for brain tumors may prove useful for preclinical drug screening against brain metastases.

## Introduction

Brain metastasis occurs in 20–40% of solid tumors, including lung cancer, breast cancer, colorectal cancer, renal cancer, and gastric cancer 1, 2. In general, brain metastasis is refractory to cytotoxic chemotherapy, and is associated with poor prognosis 3. Radiation therapy, including whole‐brain radiation and stereotactic irradiation, has the potential to control brain metastasis 4. However, stereotactic irradiation is not indicated for the treatment of a large number of brain metastases (more than 10) 5. The disease control rate, following whole‐brain irradiation, is lower than that measured following stereotactic irradiation 6. Moreover, leukoencephalopathy caused by whole‐brain irradiation may occur after a couple of years 7. Likewise, while molecular‐targeted drugs, such as the EGFR tyrosine kinase inhibitors (EGFR‐TKIs) and ALK‐TKIs, are generally effective against TKI‐naïve brain metastases, patients frequently experience acquired resistance to targeted drugs during the progression of brain metastases 8, 9. Brain metastasis is thus a so‐called sanctuary site for targeted drugs 10. Therefore, it is necessary to establish more effective treatments for controlling brain metastasis.

Animal models are useful for evaluating the efficacy of treatments at the preclinical stage 11. In particular, brain metastasis models are indispensable for the evaluation of molecular‐targeted drugs, because the effects of targeted drugs are thought to be affected by microenvironmental factors, including the blood–brain barrier (BBB) 12. For this purpose, we have recently developed an *in vivo* imaging model for brain tumors, which mimics brain metastasis using *EML4‐ALK* lung cancer cells. We further demonstrated that the second‐generation ALK‐TKI alectinib was much more effective than the first‐generation ALK‐TKI crizotinib in the *in vivo* imaging model, thus indicating the usefulness of alectinib against brain metastases induced by *EML4‐ALK* lung cancer 13. Recently, many molecular targets have been identified in various types of tumors, and the corresponding target drugs are being evaluated in clinical trials.

Here, we have assessed the production of brain tumors, using various types of human cancer cell lines, to establish a variety of brain tumor models that mimic brain metastases. We have established an *in vivo* imaging model for brain tumors for the epidermal growth factor receptor (EGFR)*‐*mutant lung cancer, the HGF‐dependent gastric cancer, and for colorectal cancer harboring the *NTRK1* gene fusion. We have further evaluated the efficacy of molecular‐targeted drugs, including the EGFR‐TKI, MET‐TKI, and the tropomyosin‐related kinase (TRK)‐TKI, in our brain tumor models, in comparison to their efficacy in extracranial tumor models, such as subcutaneous tumors, peritoneal carcinomatosis, and liver metastasis models.

## Materials and Methods

### Cell cultures and reagents

The human lung cancer cell lines H1975 14, PC‐9 15, LC319/bone 16, and PC14PE6 17, the human colorectal cancer cell lines KM12C and KM12SM 18, and the human gastric cancer cell line NUGC4 19 were used in this study. The characteristics of these cell lines are listed in Table 1. Luciferase‐transfected H1975 cells, H1975‐Luc, were provided by the JCRB Cell Bank (Osaka, Japan) 20. Luciferase‐transfected NUGC4 (NUGC4/Luc) and KM12SM (KM12SM/Luc) cells were established using the same method, as previously described 13. These cells were maintained in RPMI‐1640 medium, supplemented with 10% fetal bovine serum (FBS) and antibiotics. All cells were passaged for less than 3 months, before restarting the cultures from frozen, early‐passage stocks. Cells were regularly screened for mycoplasma contamination using the MycoAlert Mycoplasma Detection Kit (Lonza, Rockland, ME). These cells were authenticated by short tandem repeat analysis at the National Institute of Biomedical Innovation (Osaka, Japan). Gefitinib, osimertinib, crizotinib, golvatinib, and entrectinib were obtained from Selleck Chemicals (Houston, TX).

**Table 1:** Characteristics of the human cancer cell lines used in this study

	H1975	PC‐9	LC319/bone2	PC14PE6	NUGC4	KM12SM
Gene alteration	*EGFR* L858R+T790M	*EGFR* exon 19 deletion	*MET* amplification	Unknown (*VEGF* high)	HGF dependent	*TPM3‐NTRK1* fusion
Tumor type	Lung	Lung	Lung	Lung	Stomach	Colon
Incidence of brain tumor production	8/10	0/5 (5/5)^a^	2/3	3/3	18/18	15/16

a The number in parentheses indicates the incidence of leptomeningeal carcinomatosis.

### Tumor cell inoculation in severe combined immunodeficient (SHO‐Prkdc^scid^Hr^hr^) mice

Six‐week‐old SHO‐Prkdc^scid^Hr^hr^ female mice (SHO mice, Charles River, Yokohama, Japan) were used in this study. For the brain metastasis model 21, mouse scalps were sterilized with 70% ethanol, and a small hole was bored into the skull, 0.5 mm anterior and 3.0 mm lateral to the bregma, using a dental drill. Cell suspensions (1.5 × 10^5^/1.5 *μ*L) were injected into the right striatum, 3 mm below the surface of the brain, using a 10‐*μ*L Hamilton syringe with a 26G needle. The scalp was closed using an Autoclip Applier. For the subcutaneous tumor and peritoneal carcinomatosis model, tumor cells (1–3 × 10^6^/100–200 *μ*L) were implanted subcutaneously into the flanks and the peritoneal cavity of each mouse, respectively 22, 23. For the liver metastasis model, tumor cells (1 × 10^6^/50 *μ*L) were inoculated into the spleen of each mouse 18.

In the brain tumor and liver metastasis models, tumor volume was tracked in live mice using repeated noninvasive optical imaging of tumor‐specific luciferase activity, measured using the IVIS Lumina XR Imaging System (PerkinElmer, Alameda, CA), as previously described 13. The intensity of the bioluminescent signal was analyzed using the Living Image 4.0 software (PerkinElmer), by serially quantifying the peak photon flux in the selected region of interest (ROI) within the tumor. The intensity of the bioluminescent signal was corrected for the total area of the ROI and the elapsed time during which bioluminescent signals were read by the CCD camera, and this value was expressed as photons/s/cm^2^/sr. The sizes of the subcutaneous tumors and the body weights of the mice were measured twice per week, and tumor volume was calculated in mm^3^ (width^2^ × length/2).

This study was carried out in strict accordance with the recommendations of the Guide for the Care and Use of Laboratory Animals of the Japanese Ministry of Education, Culture, Sports, Science, and Technology. The protocol was approved by the Committee on the Ethics of Experimental Animals and the Advanced Science Research Center, Kanazawa University, Kanazawa, Japan (approval no. AP‐153688). All surgeries were performed on mice anesthetized with sodium pentobarbital, and efforts were made to minimize animal suffering.

### Cell viability assay

Cell viability was measured using an MTT assay. Tumor cells (2 × 10^3^ in 100 *μ*L RPMI‐1640 plus 10% FBS) were plated per well, in 96‐well plates, and incubated for 24 h. EGFR‐TKIs and/or MET‐TKIs were then added to each well, and incubation was continued for another 72 h. Cell growth was measured using the MTT solution (2 mg/mL; Sigma, St. Louis, MO), as previously described 24.

### Immunoblot analyses

Lysates were prepared using Cell Lysis Buffer (Cell Signaling, Danvers, MA). Immunoblotting was performed as previously described 13. Antibodies used in this study were as follows: anti‐EGFR, anti‐phospho‐EGFR (Tyr1068), anti‐MET, anti‐phospho MET (Tyr1234/1235), anti‐TRK, anti‐phospho TRK (Tyr490), anti‐AKT, anti‐phospho‐AKT (Ser473), and anti‐*β*‐actin (13E5) antibodies (each used at a 1:1000 dilution; Cell Signaling Technology, Danvers, MA). Additional antibodies were also used, including the anti‐human/mouse/rat extracellular signal‐regulated kinase ERK1/ERK2 (0.2 *μ*g/mL) and the anti‐phospho‐ERK1/ERK2 (T202/Y204) (0.1 *μ*g/mL) from R&D Systems. The immunoreactive bands were visualized using the SuperSignal West Dura Extended Duration Substrate, an enhanced chemiluminescent substrate (Pierce Biotechnology, Rockford, IL). Each experiment was performed independently at least three times.

### Immunohistochemical staining

Sections with a thickness of 5 *μ*m were deparaffinized in xylene and rehydrated in decreasing concentrations of ethanol. Following antigen retrieval, the endogenous peroxidase activity was blocked using a 3% aqueous H_2_O_2_ solution for 10 min. The sections were treated with 5% normal horse serum and then incubated with the following primary antibodies: anti‐MET, anti‐phospho‐Met (Y1234/Y1235), anti‐TRK‐A, anti‐phospho TRK‐A (Tyr490) (Santa Cruz Biotechnology). After probing with species‐specific biotinylated secondary antibodies, sections were allowed to react for 30 min with an avidin–biotin–peroxidase complex (ABC), using a Vectastain ABC kit (Vector Laboratories, Burlingame, CA). The DAB (3,3′‐diaminobenzidine tetrahydrochloride) Liquid System (Dako Cytomation) was used for detection.

## Results

### Brain tumor production using human cancer cell lines

We inoculated six different human cancer cell lines into the brain parenchyma of SHO mice, and evaluated tumor production. H1975, NUGC4, KM12SM, LC319/bone2, and PC14PE6 cell lines produced tumors in the brain parenchyma, while PC‐9 cells developed leptomeningeal carcinomatosis, without detectable tumors in the brain parenchyma (Table 1, Fig. S1).

### The effect of kinase inhibitors on the viability of human cancer cell lines *in vitro*

**Figure 1:**
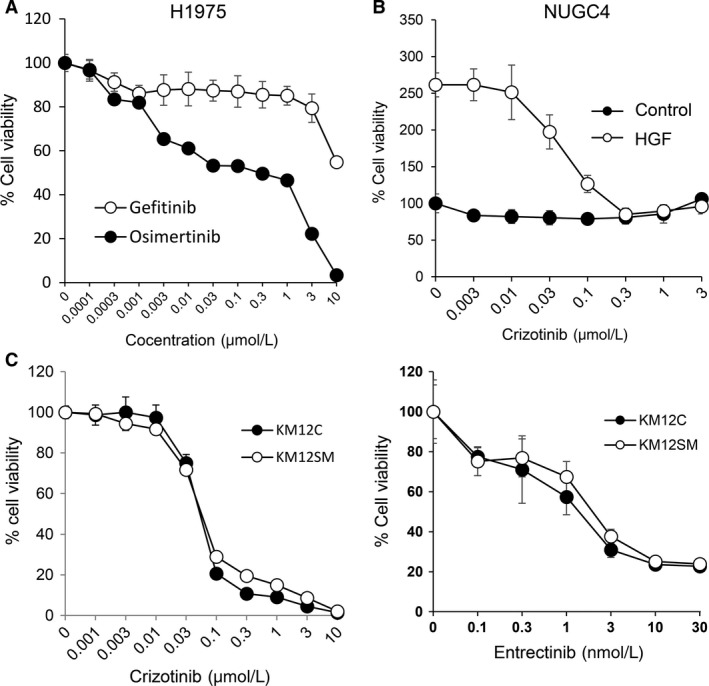
The effect of kinase inhibitors on the viability of human cancer cell lines *in vitro*. (A) H1975 cells treated with osimertinib or gefitinib for 72 h. (B) NUGC4 cells treated with crizotinib, in the presence or absence of HGF, for 72 h. (C) KM12C and KM12SM cells treated with crizotinib or entrectinib for 72 h. Cell viability was determined using the MTT assay. The data are representative of three independent experiments, showing similar results. The bars indicate SDs of quadruplicate cultures.

We next evaluated the susceptibility of five human cancer cell lines, with the potential to produce brain tumors, to targeted drugs *in vitro*. Human lung adenocarcinoma H1975 cells, harboring an *EGFR‐*L858R‐sensitive mutation or an *EGFR‐*T790M‐resistant mutation, were resistant to the first‐generation EGFR‐TKI gefitinib, but were sensitive to the third‐generation EGFR‐TKI osimertinib (Fig. 1A). The proliferation of NUGC4 human gastric cancer cells was stimulated by HGF 23. While crizotinib, that inhibits the activities of multiple kinases including ALK, MET, and TRK‐A, did not affect the viability of basal cells, it suppressed the HGF‐induced proliferation in a dose‐dependent manner (Fig. 1B), as we have previously reported 23.

**Figure 2:**
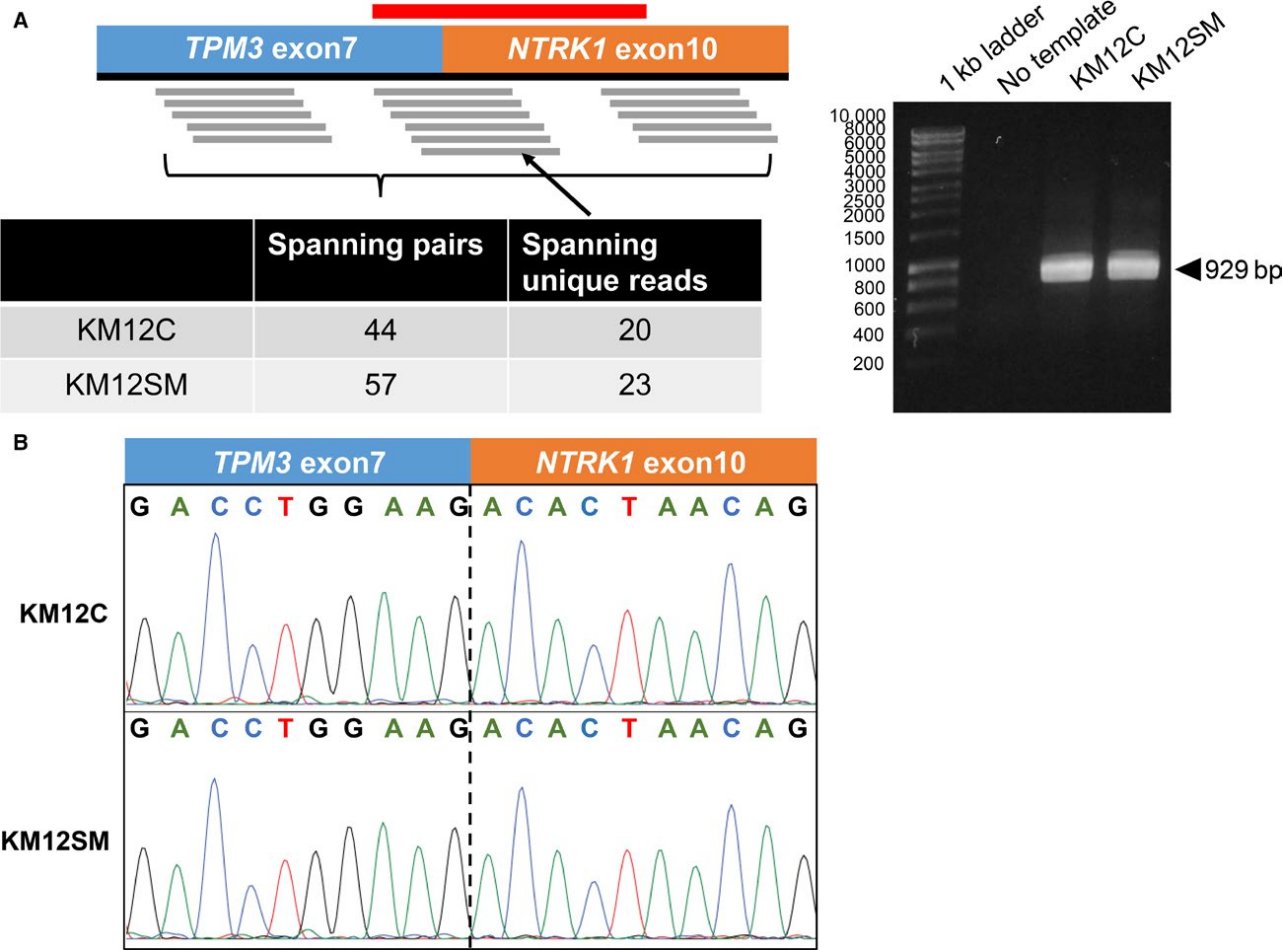
KM12C and KM12SM cells harbor the *TPM3‐NTRK1* gene fusion. (A) Schematic representation of RNA sequencing reads, supporting the presence of *TPM3‐NTRK1* fusion transcripts. The FusionCatcher software was used to count the number of paired‐end reads that supported the fusion transcripts (“Spanning pairs”) and that were mapped on the fusion junction (“Spanning unique reads”), respectively. The *red‐colored transverse line* illustrates the RT‐PCR target region, encompassing the *TPM3‐NTRK1* fusion junction. (B) RT‐PCR, followed by agarose gel electrophoresis, confirmed the presence of *TPM3‐NTRK1* fusion transcripts in KM12C and KM12SM cells. (C) Sanger sequencing of the RT‐PCR products, identified the fusion junctions of the *TPM3‐NTRK1* fusion transcripts in the two cell lines.

The KM12SM cell line is a highly liver‐metastatic variant of KM12C colon cancer cells, obtained after repeated *in vivo* selection 18. A recent study reported that KM12C cells are positive for the *TPM3‐NTRK1* gene fusion, and sensitive to crizotinib, which inhibits TRK‐A 25. We performed RNA sequencing and RT‐PCR, and confirmed that both KM12C and KM12SM cells contained the *TPM3‐NTRK1* gene fusion (Fig. 2). In addition, crizotinib and entrectinib, which inhibit TRK‐A, suppressed the viability of KM12SM and KM12C cells, in a dose‐dependent manner (Fig. 1C). In addition, KM12SM and KM12C cells displayed similar sensitivities to other MET inhibitors, such as golvatinib and GW441756 (Fig. S2). Conversely, LC319‐bone2 and PC14PE6 cells were refractory to the tested targeted drugs (Fig. S3). These results indicate that H1975, NUGC4, and KM12SM cells are sensitive to the corresponding targeted drugs *in vitro*.

### The effect of kinase inhibitors on signal transduction in human cancer cell lines *in vitro*

**Figure 3:**
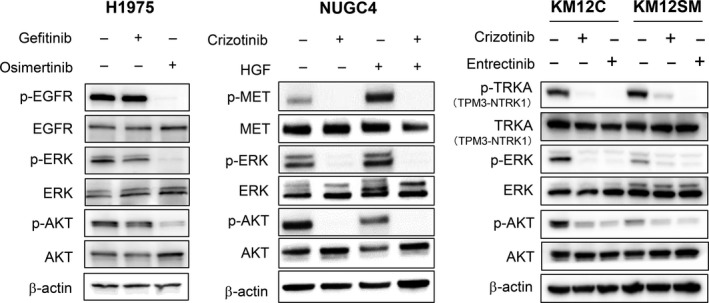
The effect of kinase inhibitors on signal transduction in human cancer cell lines *in vitro*. H1975 cells were treated with osimertinib (1 *μ*mol/L) for 2 h. NUGC4 cells were treated with crizotinib (1 *μ*mol/L) for 2 h, and then stimulated with HGF (50 ng/mL) for 10 min. KM12C and KM12SM cells were treated with crizotinib (1 *μ*mol/L) or entrectinib (1 *μ*mol/L) for 2 h. Immunoblots of cell lysates from these treated cell lines are shown. The data are representative of three independent experiments, showing similar results.

We next examined the expression and phosphorylation of target proteins, and those of their downstream molecules. In H1975 cells, osimertinib inhibited the phosphorylation of EGFR and that of the downstream AKT and ERK, while gefitinib failed to do so (Fig 3). In NUGC4 cells, crizotinib inhibited the constitutive phosphorylation of MET and that of the downstream AKT and ERK. HGF enhanced the phosphorylation of MET and that of the downstream AKT and ERK, while crizotinib abolished this enhanced phosphorylation triggered by HGF.

In KM12C and KM12SM cells, a 70‐kDa TRK‐A protein compatible in size with the product of the *TPM3‐NTRK1* gene fusion was detected, and TRK‐A was constitutively phosphorylated. Both crizotinib and entrectinib inhibited the phosphorylation of TRK‐A and that of the downstream AKT and ERK in KM12C and KM12SM cells.

### Osimertinib inhibited the progression of brain tumors and subcutaneous tumors produced by H1975 cells

**Figure 4:**
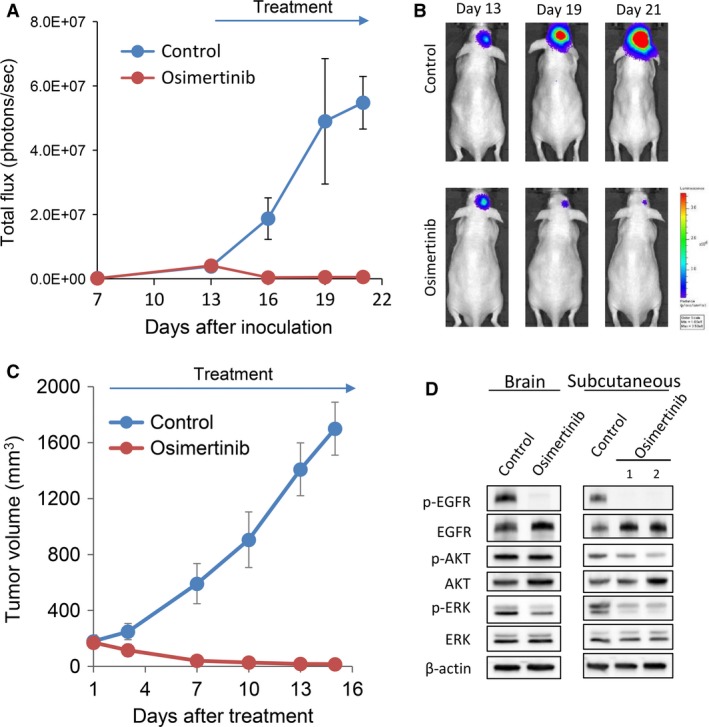
Osimertinib inhibits the progression of brain and subcutaneous tumors produced by H1975 cells. (A) H1975‐Luc cells were inoculated into the brain of SHO mice. The mice were treated with control (*N* = 3) or osimertinib (25 mg/kg per day) (*N* = 3) from day 8 to day 24. Bioluminescence was determined twice a week. Data represent the means ± SEs. (B) Representative images of mice from panel A are shown. (C) H1975‐Luc cells were inoculated subcutaneously into SHO mice. The mice were treated with control (*N* = 3) or osimertinib (25 mg/kg per day) (*N* = 3), when tumor volume became larger than 150 mm^3^, for 15 days. (D) H1975‐Luc cells were inoculated into the brain, or subcutaneously in SHO mice. After the growth of tumors, the mice were treated with control (*N* = 1) and osimertinib (25 mg/kg per day) (*N* = 2) for 4 days. Four hours after the final administration of osimertinib, mice were killed and tumors were harvested. The expression of the indicated proteins in tumor lysates are shown in immunoblots.

We next explored the effect of targeted drugs in our brain tumor model by comparing with extracranial tumor models, using H1975, NUGC4, and KM12SM cells. In H1975 cell models, osimertinib (25 mg/kg) inhibited the progression of brain tumors and that of subcutaneous tumors (Fig. 4A). Western blot analyses of *in vivo*‐treated tumors revealed that treatment with osimertinib efficiently inhibited the phosphorylation of EGFR and that of its downstream molecule, ERK, in both brain tumor and subcutaneous tumor models (Fig. 4B).

### Crizotinib inhibited the progression of brain tumors and that of peritoneal carcinomatosis produced by NUGC4 cells

**Figure 5:**
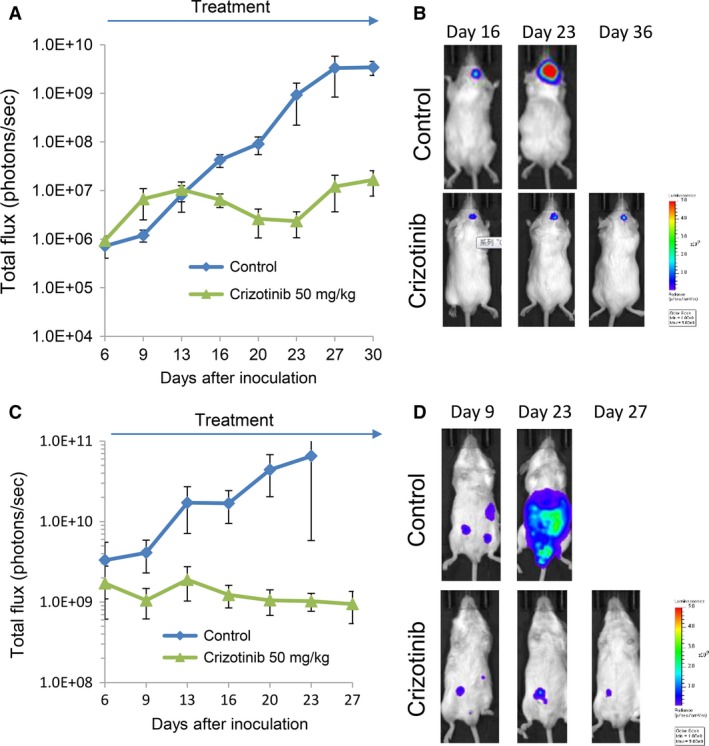
Crizotinib inhibits the progression of brain tumors and that of peritoneal carcinomatosis produced by NUGC4/Eluc cells. NUGC4/Eluc cells were inoculated into the brain (A, *N* = 5/group) or peritoneal cavity (C, *N* = 6/group) of SHO mice. The mice were treated with control or crizotinib (50 mg/kg per day) from day 7 to day 28. Bioluminescence was determined twice a week. Data represent the means ± SEs. Representative images for A and C are shown in B and D, respectively.

In NUGC4 cell models, we evaluated the effect of crizotinib (50 mg/kg), since we have previously reported that 25 mg/kg and 50 mg/kg of crizotinib inhibited the progression of peritoneal carcinomatosis produced by NUGC4 cells 23, and that of brain tumors produced by EML4‐ALK‐positive A925L lung cancer cells 15, respectively. Consistent with these findings, crizotinib (50 mg/kg) delayed the progression of brain tumors (Fig. 5A and B) and that of peritoneal carcinomatosis (Fig. 5C and D). We have previously reported that treatment with crizotinib inhibited MET phosphorylation in peritoneal tumors produced by NUGC4 cells, demonstrating the proof of concept underlying the use of crizotinib as a MET inhibitor 23. In this study, we have further discovered that treatment with crizotinib inhibited MET phosphorylation in brain tumors produced by NUGC4 cells (Fig. S4), as determined by immunohistochemistry. Crizotinib is a well‐known substrate of a component of the blood–brain barrier, although it poorly penetrates the brain 26. These results, however, clearly indicate that crizotinib possesses an antitumor activity against both brain tumors and peritoneal carcinomatosis produced by NUGC4 cells.

### Entrectinib, but not crizotinib, inhibited the progression of brain tumors produced by KM12SM cells

**Figure 6:**
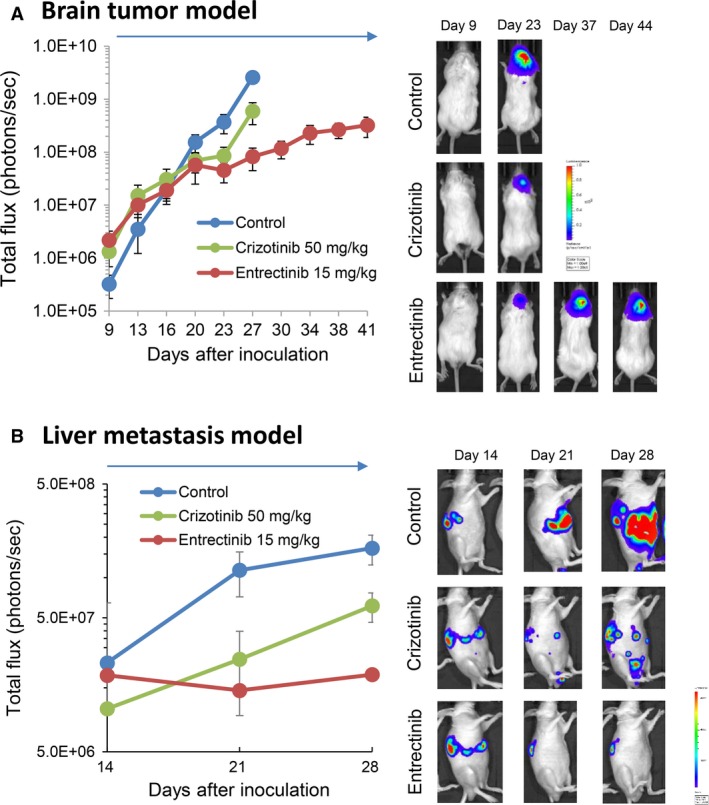
Entrectinib, but not crizotinib, inhibits the progression of brain tumors produced by KM12SM/Eluc cells. KM12SM/Eluc cells were inoculated into the brain (A, *N* = 5/group) or the peritoneal cavity (C, *N* = 6/group) of SHO mice. The mice were treated with control, crizotinib (50 mg/kg per day), or entrectinib (15 mg/kg per day) from day 7 to 28. Bioluminescence was determined twice a week. Data represent the means ± SEs. Representative images for A and C are shown in B and D, respectively.

In KM12SM cell models, we evaluated the effect of crizotinib (50 mg/kg) and entrectinib (15 mg/kg) in brain tumor models and liver metastasis models. Continuous daily treatment with crizotinib slightly delayed the progression of brain tumors (Fig. 6A), but four out of six mice treated with crizotinib died by day 27 due to the progression of brain tumors. Continuous daily treatment with entrectinib more discernibly delayed the progression of brain tumors. Thus, all mice treated with entrectinib survived until day 41. On the other hand, in the liver metastasis model, we treated mice with crizotinib or entrectinib for 14 days, due to a limited supply of drugs. Treatment with crizotinib for 14 days discernibly delayed the development of liver metastases (Fig. 6B). Moreover, treatment with entrectinib for 14 days inhibited liver metastases more noticeably than crizotinib. These results indicate that crizotinib may possess antitumor activities against liver metastases, but not against brain metastases. Furthermore, entrectinib displayed a superior activity in inhibiting the progression of KM12SM tumors in the liver and brain, when compared with that of crizotinib.

## Discussion

In this study, we have established *in vivo* imaging models for brain tumors that mimic brain metastases for *EGFR*‐mutant lung cancer, HGF‐dependent gastric cancer, and *NTRK1*‐fusion‐positive colon cancer. Moreover, we examined the efficacy of molecular‐targeted drugs in these brain tumor models, by comparison with extracranial tumor models. We found that: (1) even when the same cell line was inoculated, the drug efficacy could be different between the brain tumor model and the extracranial tumor model (in the case of KM12SM cells), and (2) the same drug, harboring a multikinase inhibitory activity, could show different efficacies in the brain tumor models that contained different drivers (in the case of crizotinib). Since these phenomena can be observed in the clinical practice, our brain tumor models may prove useful for the screening of highly efficient targeted drugs against brain metastases.

*EGFR*‐activating mutations, such as the deletion of exon 19, and the L858R point mutation in exon 21, are detected almost exclusively in lung adenocarcinoma 27, 28. There are considerable ethnic differences with respect to the incidence of *EGFR* mutations in lung adenocarcinoma between East Asian individuals and others (50–60% vs. 8–10% for lung cancer in East Asian and Caucasian individuals, respectively) 27. *EGFR*‐mutant lung cancer frequently leads to the development of brain metastases, compared with non‐small‐cell lung cancer (NSCLC) containing a wild‐type copy of *EGFR* 29. The *EGFR*‐T790M mutation is detectable in 50–60% of *EGFR*‐mutant lung cancers, whose extracranial tumor lesions have acquired resistance to first‐generation EGFR‐TKIs 30. However, there are few reports assessing the incidence of *EGFR*‐T790M mutations in brain metastases with acquired resistance to EGFR‐TKIs. On the other hand, the *NTRK1* gene fusion occurs with low incidence in several solid tumors, including NSCLC, colorectal cancer, intrahepatic cholangiocarcinoma, papillary thyroid cancer, spitzoid neoplasms, glioneuronal tumors, and sarcomas 25, 31, 32, 33, 34, 35, 36. Few reports exist on the incidence of brain metastasis in *NTRK1*‐fusion‐positive cancers. Moreover, KM12SM cells exhibited a high potential for developing tumors after intracranial inoculation, and brain metastases after internal carotid artery inoculation 37. Thus, the *NTRK1* gene fusion might be associated with a high metastatic potential in the brain. Further studies containing larger cohorts are necessary to clarify the clinical characteristics of *NTRK1*‐fusion‐positive cancers.

Osimertinib has been approved for the treatment of *EGFR*‐mutated lung cancer that has acquired EGFR‐TKI resistance due to the T790M mutation. Osimertinib is reported to achieve a greater penetration of the mouse blood–brain barrier than other EGFR‐TKIs, including gefitinib, afatinib, and rociletinib (CO‐1686), although osimertinib is the substrate of the P‐glycoprotein, which is expressed as a component of the BBB 38. Several reports have shown that both the intracranial and extracranial responses to osimertinib, in patients in whom the T790M mutation was detected, lead to the formation of extracranial tumor lesions 39. Accordingly, osimertinib displayed a remarkable efficacy in both the brain and the subcutaneous tumor models containing T790M‐positive H1975 cells. These results suggest that our models, containing H1975 cells, may be useful for the screening of inhibitors with activity against both intracranial and extracranial lesions.

Crizotinib is the first approved drug for *ALK*‐fusion‐positive NSCLC. The drug is active against multiple kinases, including MET, TRK, and AXL 40. In *in vitro* experiments, crizotinib abolished the growth of NUGC4 cells stimulated by the exogenously added human recombinant HGF. Crizotinib also decreased the viability of KM12SM cells, presumably via the suppression of TRK‐A phosphorylation. Moreover, treatment with crizotinib remarkably inhibited the progression of peritoneal carcinomatosis, and that of brain tumors in the NUGC4 cell model, and the progression of liver metastases in the KM12SM cell model. While HGF is believed to induce species‐specific biological activities 41, we have recently reported that mouse HGF is efficient, but slightly less effectively than human HGF, in stimulating the proliferation of NUGC4 cells *in vitro* 23. Therefore, we speculate that the mouse‐derived HGF stimulated the growth of NUGC4 cells, and that crizotinib inhibited the progression of peritoneal carcinomatosis and brain tumors *in vivo*. Crizotinib is a well‐known substrate of the P‐glycoprotein, and shows low penetration into the brain 42. Although crizotinib shows moderate activity against brain metastases in TKI‐naïve *ALK‐*fusion‐positive lung cancers, a recurrence in CNS lesions is frequently observed at the acquisition of resistance 42, indicating the lower efficacy of crizotinib against brain metastases, compared to its efficacy against extracranial tumor lesions. It is interesting to note that treatment with crizotinib at the same dose (50 mg/kg) inhibited the progression of brain tumors promoted by NUGC4 cells, but not by KM12SM cells, in our *in vivo* imaging models. While the reason for this discrepancy in these two cell line models is unknown at present, a low concentration of crizotinib may be sufficient for blocking the HGF/MET signal in NUGC4 cells, but not for the suppression of TRK‐A signaling in KM12SM cells. Since no phosphorylated TRK‐A specific antibody was available for immunohistochemistry, we could not perform histological examinations to test the effect of targeted drugs on TRK‐A phosphorylation. The development of an antibody specific to phosphorylated TRK‐A for immunohistochemistry is warranted for the clarification of the molecular mechanism that determines the sensitivity to targeted drugs in various organs, including the brain.

Entrectinib is also an inhibitor of multiple kinases, including ALK, ROS‐1, and TRK‐A 43, and its efficacy is being evaluated in several clinical trials 44, 45, 46. Entrectinib is reported to highly penetrate the BBB, and several case reports have demonstrated a rapid response in brain metastases and extracranial tumor lesions of patients with ALK‐fusion‐positive NSCLC 35. Based on these observations, treatment with entrectinib considerably inhibited the development of KM12SM cells in both the brain tumor model and the liver metastasis model. Although *NTRK1*‐fusion‐positive tumors are rare, clinical trials with patients selected for *NTRK1* gene fusions are ongoing for the evaluation of the efficacy of entrectinib.

The molecular mechanisms underlying targeted drug resistance are actively investigated utilizing clinical specimens, obtained by re‐biopsy. Recent studies suggest that the mechanism of targeted drug resistance in brain lesions is often different from that in extracranial tumor lesions, presumably due to different pharmacokinetics between the brain and the extracranial organs 9. Moreover, the re‐biopsy of brain lesions at the acquisition of targeted drug resistance is not easy in practice, because of its invasiveness. Our brain tumor models, containing different tumor cell lines with different driver oncogenes, may prove useful for investigating the molecular mechanisms underlying targeted drug resistance in brain metastases. In fact, we have successfully induced the acquired resistance to osimertinib and entrectinib in brain tumor models using H1975 cells and KM12SM cells, respectively. We are now analyzing the molecular mechanisms of acquired resistance by utilizing cell lines established from brain lesions in these models. Recent progress of imaging technology allow us intravital imaging of the brain tumors 47. Surgical orthotopic implantation of tumors 48 combined with such intravital imaging may further improve the clinical relevance of the xenograft models for the evaluation of anti‐brain tumor efficacy of targeted drugs.

In summary, we have established an *in vivo* imaging system for brain tumor models of *EGFR*‐mutant lung cancers, HGF‐dependent gastric cancers, and *NTRK1*‐fusion‐positive colon cancers. The findings of this study suggest that a careful screen of the targeted drugs that are effective against brain tumors should be performed, by utilizing clinically relevant animal models, and that our *in vivo* imaging brain tumor models may prove useful for such a screen.

## Conflict of Interest

Seiji Yano obtained speakers fees and research grants from AstraZeneca.

## Supporting information


**Fig. S1.** Morphology of brains tumors produced by human cancer cell lines in SHO mice.

**Fig S2.** The sensitivity of KM12C and KM12SM cells to MET inhibitors *in vitro*.

**Fig S3.** The effect of kinase inhibitors on the viability of LC319/bone2 and PC14PE6.

**Fig S4.** The effect of crizotinib on MET phosphorylation, in brain tumors produced by NUGC4/Eluc cells.
