# Supplementary figures and images for: In vivo imaging xenograft models for the evaluation of anti‐brain tumor efficacy of targeted drugs
Source: Cancer Med. 2017 Nov 10;6(12):2972–83. doi: 10.1002/cam4.1255 (PMC5727243; doi:10.1002/cam4.1255)

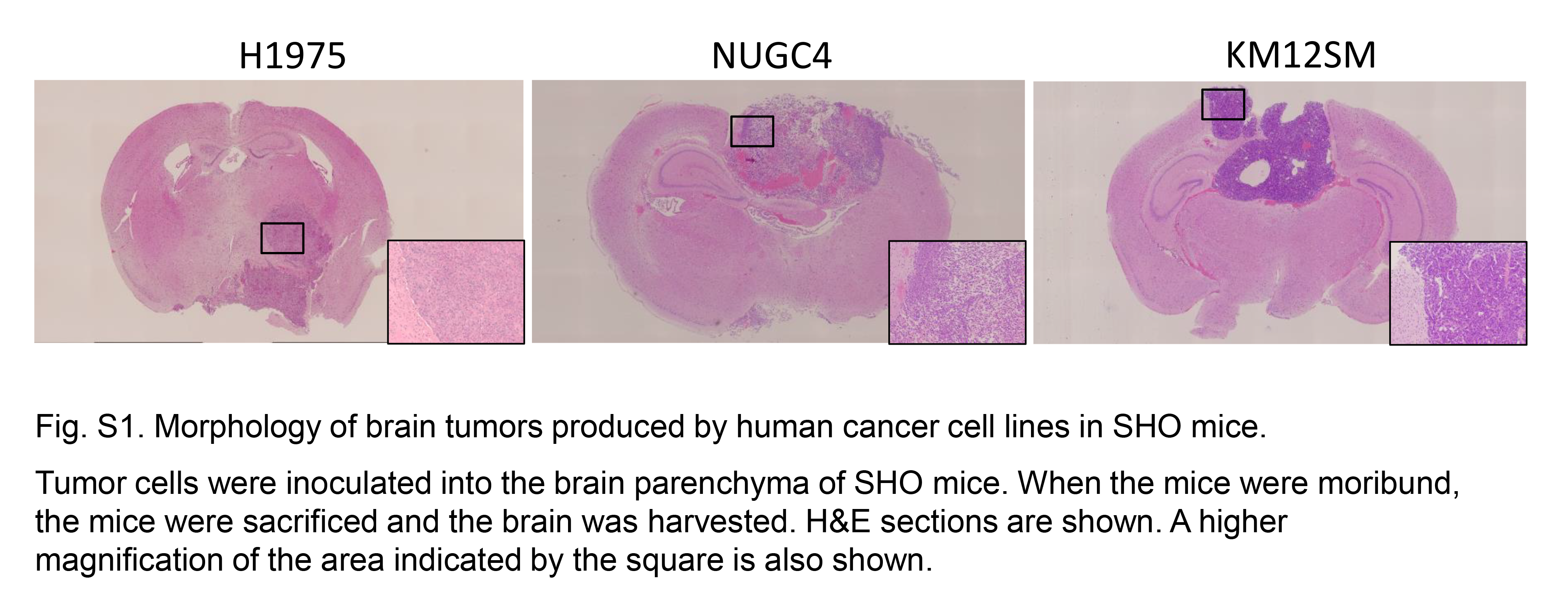

Supplement: Supplementary file 1 — Fig. S1. Morphology of brains tumors produced by human cancer cell lines in SHO mice. [file CAM4-6-2972-s001.tif]

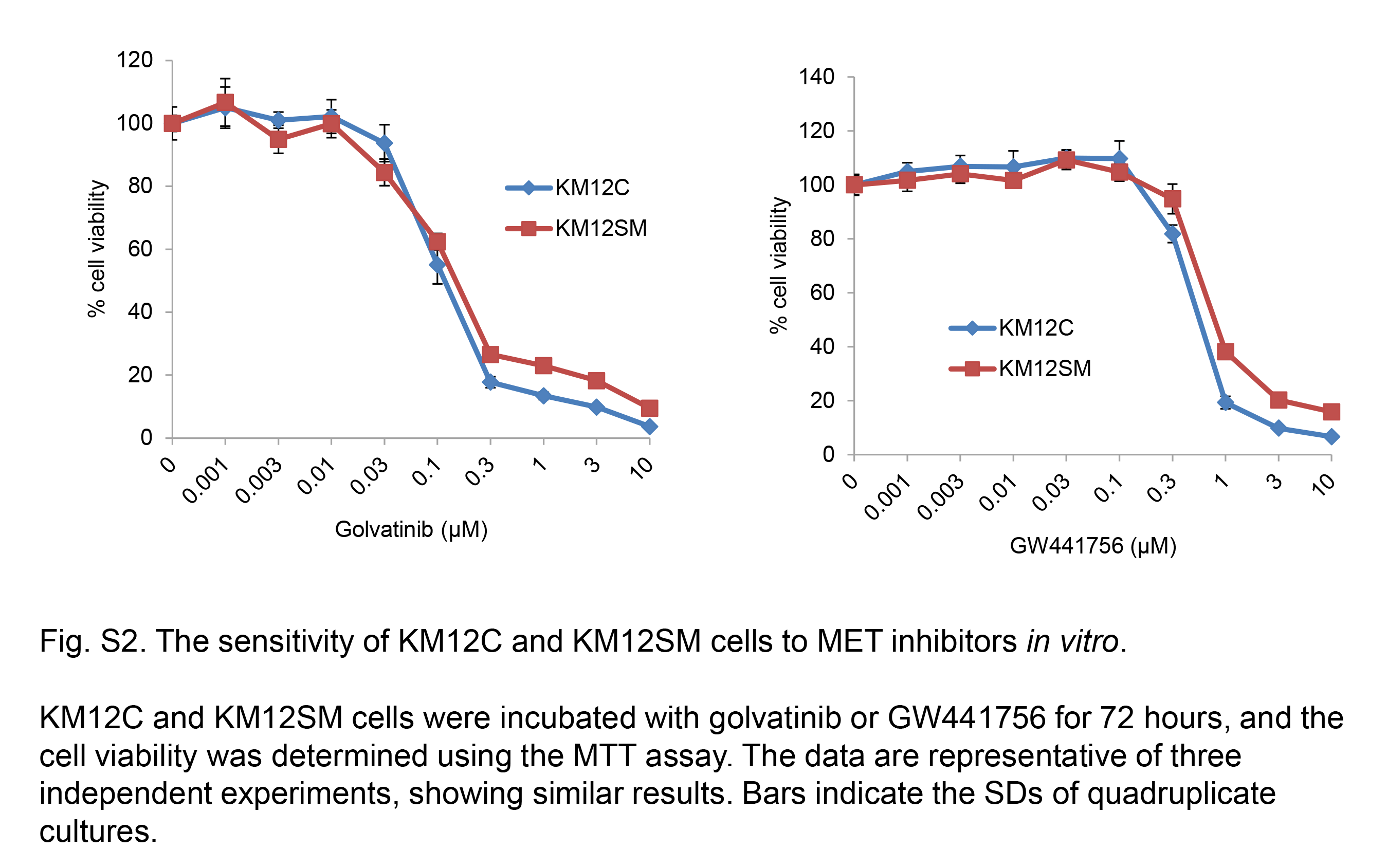

Supplement: Supplementary file 2 — Fig S2. The sensitivity of KM12C and KM12SM cells to MET inhibitors in vitro. [file CAM4-6-2972-s002.tif]

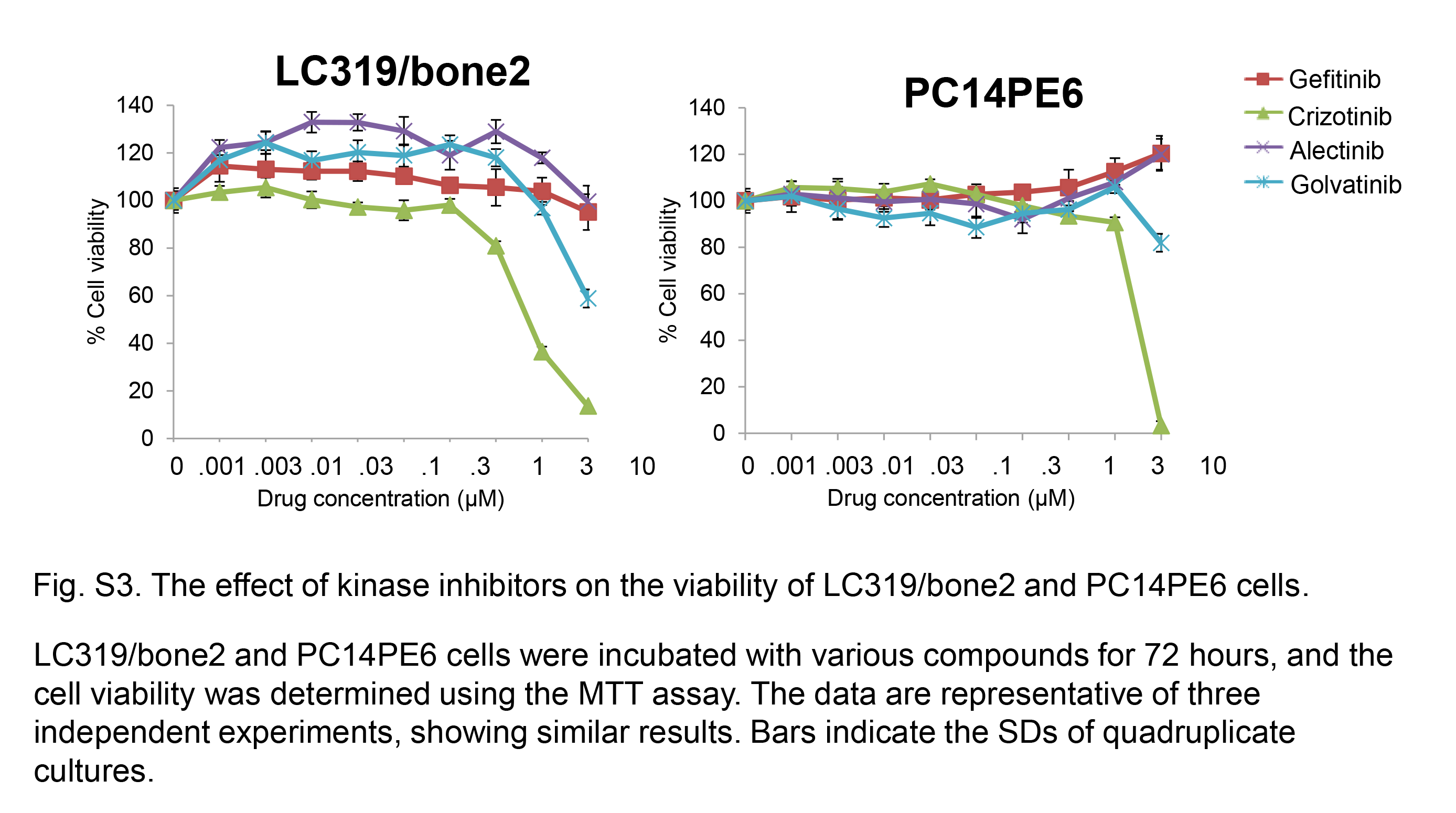

Supplement: Supplementary file 3 — Fig S3. The effect of kinase inhibitors on the viability of LC319/bone2 and PC14PE6. [file CAM4-6-2972-s003.tif]

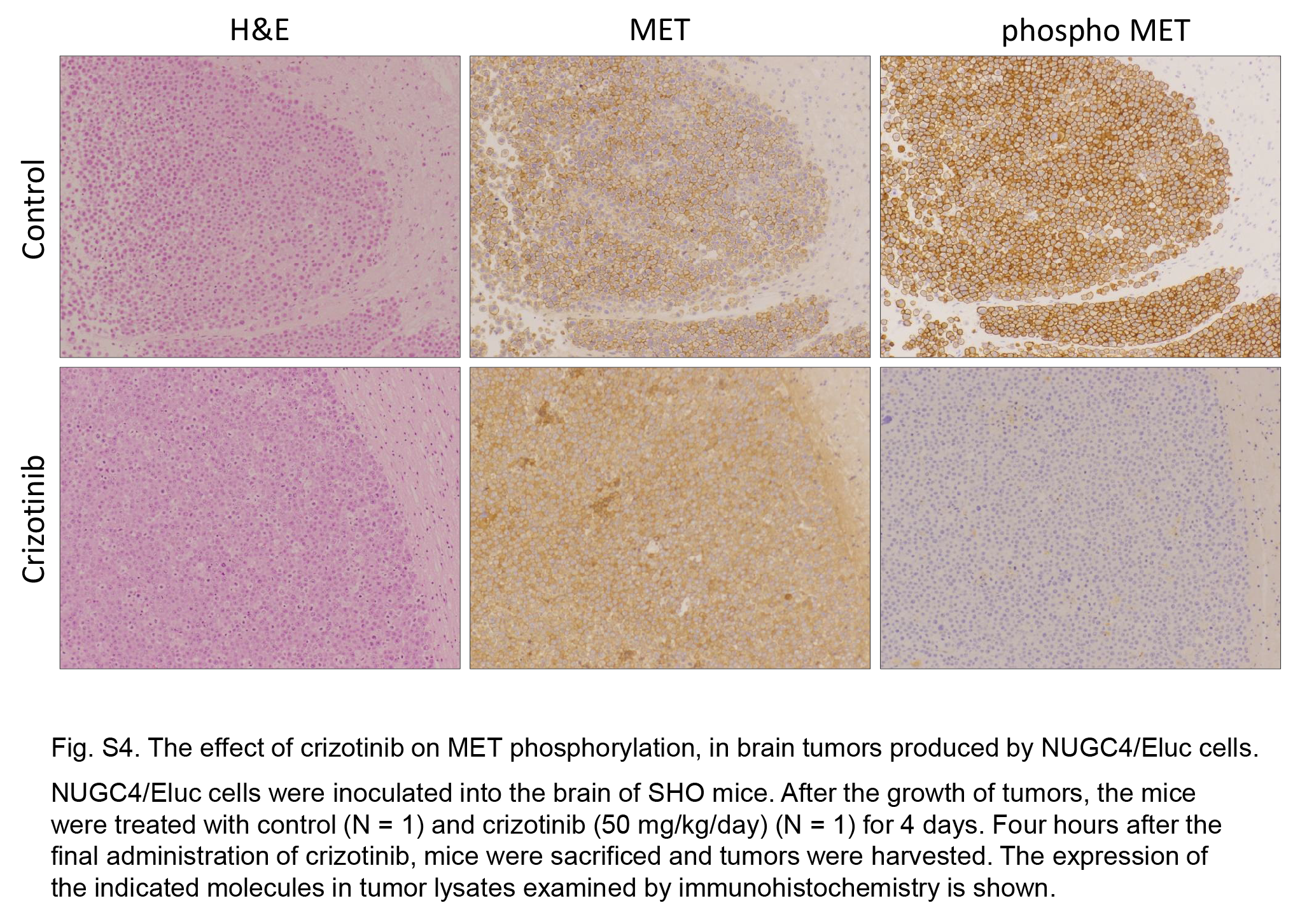

Supplement: Supplementary file 4 — Fig S4. The effect of crizotinib on MET phosphorylation, in brain tumors produced by NUGC4/Eluc cells. [file CAM4-6-2972-s004.tif]
